# Negative Out-of-Plane Electromechanical Response in Nonpiezoelectric van der Waals Layered Materials Encapsulated by Monolayer Boron Nitride

**DOI:** 10.1021/acs.jpclett.5c02427

**Published:** 2025-10-09

**Authors:** Qiong Liu, Vijay Kumar Choyal, Han Hu, Timon Rabczuk, Xiaoning Jiang, Xiaoying Zhuang

**Affiliations:** † Institute of Photonics (IOP), Faculty of Mathematics and Physics, 26555 Leibniz University Hannover, Hannover 30167, Germany; ‡ Laboratory of Nano and Quantum Engineering (LNQE), Leibniz University Hannover, Hannover 30167, Germany; § Institute of Structural Mechanics, 26597 Bauhaus University, Weimar 99423, Germany; ∥ Department of Mechanical and Aerospace Engineering, 6798 North Carolina State University, Raleigh, North Carolina 27695-7910, United States; ⊥ Department of Geotechnical Engineering, College of Civil Engineering, Tongji University, Shanghai 200092, China; # Cluster of Excellence PhoenixD (Photonics, Optics and Engineering - Innovation Across Disciplines), Hannover 30167, Germany

## Abstract

The negative out-of-plane piezoelectric response is a rare phenomenon in nonpiezoelectric van der Waals (vdW) materials. In this study, we present a straightforward method to manipulate the sign of the out-of-plane piezoelectric responses of vdW centrosymmetric materials by creating vdW heterostructures. Piezoresponse force microscopy (PFM) results indicate that the out-of-plane piezoresponse of WSe_2_ nanoflakes changes from positive to negative when capped with a monolayer of hexagonal boron nitride (h-BN). Based on the Kelvin probe force microscopy (KPFM) results and first-principles calculations, the charge transfer is examined to occur between WSe_2_ and h-BN, resulting in a built-in electric field (EF). The observed negative piezoresponse in the WSe_2_/BN heterostructures can be attributed to the built-in EF and weak interlayer vdW forces. Our work enhances the understanding of the electromechanical performances of vdW layered heterostructures and may provide a new method for designing novel vdW material-based devices utilizing electromechanical properties.

## Graphical abstract

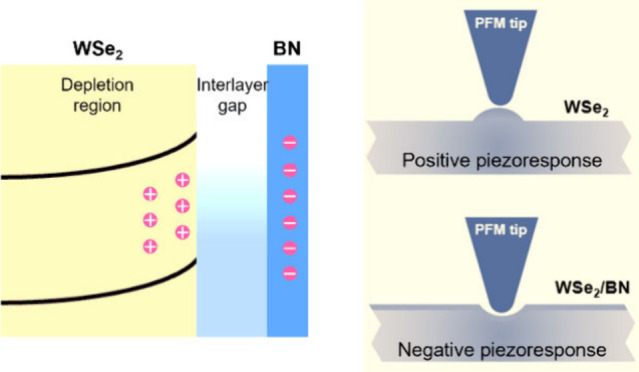

Electromechanical properties of materials refer to their ability to convert mechanical energy into electrical energy or vice versa. These materials are essential for various contemporary and future applications, including self-powered electronics, actuators, and sensors.^1–4^ Among the different types of electromechanical effects, piezoelectricity occurs only in materials that lack a center of symmetry. Typically, the longitudinal piezoelectric coefficients (*d*_33_) are positive, indicating that a tensile strain results in increased polarization or that the material extends along the external electric field (EF).^5^ In contrast, materials with negative longitudinal piezoelectricity (NLP) will contract in the direction of the applied EF. However, NLP in single-phase materials has been considered quite rare and thus has received less experimental attention. Nevertheless, NLP has been theoretically predicated in various materials, such as III–V zinc blende semiconductors, hexagonal ABC ferroelectrics, and some low-dimensional van der Waals (vdW) materials.^6–10^

Recently, NLP was experimentally observed in single-phase ferroelectric polymer poly­(vinylidene fluoride) (PVDF) and vdW ferroelectric CuInP_2_S_6_.^9,11^ Theoretical calculations have revealed that this unusual phenomenon results from the interplay between two competing factors: the “clamped-ion” and “internal-strain” terms; when the contribution from the negative “clamped-ion” term outweighs that from the positive “internal-strain” term or when the positive “clamped-ion” term is unable to compete with the negative “internal-strain” term, the overall piezoresponse becomes negative. Due to the “lag of Wannier center” effect, low-dimensional vdW materials with out-of-plane piezoelectricity usually have negative “clamped-ion” terms. Furthermore, mechanical strain that may induce structural changes can be accommodated by low-dimensional vdW materials through their interlayer gaps, making the positive “internal-strain” term negligible. In this context, NLP is anticipated to be prevalent in low-dimensional vdW materials with preexisting out-of-plane polarization beyond vdW ferroelectrics.^5,11^

It is well-known that bulk vdW crystals belonging to the *D*_6*h*_ point group, such as bulk hexagonal transition metal dichalcogenides (2H-TMDs), are centrosymmetric and therefore exhibit no intrinsic piezoelectricity.^12,13^ Only in-plane piezoelectricity exists in 2H-TMDs with odd-number layers due to the lack of an inversion center. However, various TMDs with out-of-plane mirror symmetry, such as MoS_2_, MoSe_2_, WSe_2_, and WS_2_, have demonstrated an effective out-of-plane coefficient *d*_33,eff_ when measured using piezoresponse force microscopy (PFM).^14^ Flexoelectricity offers the vdW material family without structural asymmetry great potential in applications using electromechanical properties, such as the flexo-photovoltaic effect observed in MoS_2_.^15^ The *d*_33,eff_ coefficient resulting from flexoelectricity is usually positive, leading to a local volume expansion under PFM measurements (Figure 1a–c). Negative *d*_33,eff_ coefficients have also rarely been reported in low-dimensional nonpiezoelectric vdW materials. There has been research showing that the magnitude of out-of-plane piezoresponse of TMDs nanoflakes can be tuned through heterostructure engineering.^12,16^ For instance, our previous results showed that the effective positive out-of-plane piezoelectricity of MoS_2_ nanoflakes was significantly enhanced by forming MoS_2_/BN heterostructures.^12^ It is natural to ask whether the sign of *d*_33,eff_ of TMDs would be changed with the formation of vdW heterostructures. Revealing this will provide a deeper understanding of NLP.

**Figure 1:**
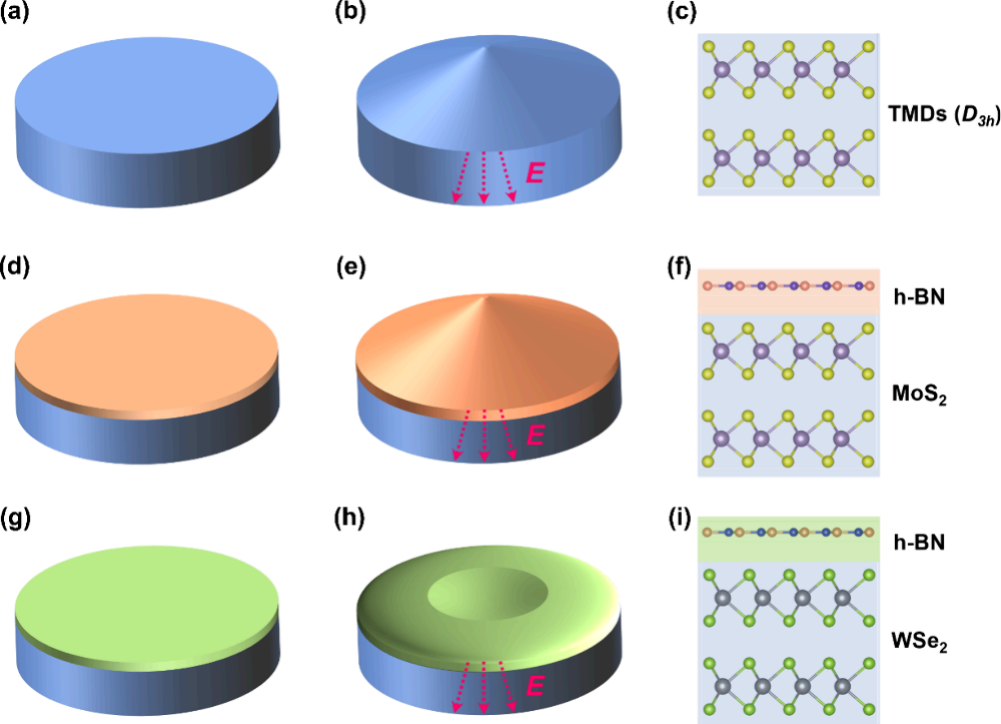
Schematic of the electromechanical properties for low-dimensional vdW materials. Illustrations of (a–c) local volume expansion induced by flexoelectricity in TMDs under an uneven EF (EF gradient), (d–f) positive longitudinal piezoelectricity in MoS_2_/BN, and (g–i) NLP in WSe_2_/BN.

In this study, we used PFM to examine the out-of-plane electromechanical properties of vdW heterostructures formed by stacking monolayer hexagonal boron nitride (h-BN) on WSe_2_ nanoflakes. Both the pristine WSe_2_ nanoflakes and monolayer h-BN exhibited positive *d*_33,eff_ coefficients. However, unlike MoS_2_/BN, which shows an enhanced positive *d*_33,eff_ coefficient (Figure 1d–f),^12^ our findings in this work revealed that the as-prepared WSe_2_/BN heterostructures had a local volume contraction, indicating a negative *d*_33,eff_ coefficient, as illustrated in Figure 1g–i. Through molecular dynamics (MD) simulations and density functional theory (DFT) calculations in conjunction with our experimental results, we explain that the negative effective out-of-plane piezoelectricity of WSe_2_/BN arises from the built-in EF near the interface, which is induced by charge transfer between WSe_2_ and BN.

Figure S1 presents typical AFM topography images and optical microscopy images of the as-prepared WSe_2_ nanoflakes and WSe_2_/BN heterostructures. The electromechanical responses of the samples were measured using PFM, as illustrated in Figure S2a. An alternating voltage (AC) voltage with a frequency of 60 kHz, far from the resonance frequency of the tip–sample system (see Figure S2b), was applied between the conductive AFM tip and the gold substrate. This application created EFs across the samples, inducing mechanical deformations in the materials that were then detected by a position-sensitive photodiode.

PFM amplitude images (Figure 2a) of the WSe_2_ nanoflake (shown in the inset of Figure 2a) demonstrate a progressively enhanced contrast. This indicates a consistent increase in the piezoresponse of the nanoflake with the applied AC voltage. The crystal structure of WSe_2_ nanoflakes belongs to the *D*_3*h*_ point group, which does not exhibit an out-of-plane piezoelectricity. Furthermore, the influence of electrostatic forces on the piezoresponse is negligible, as illustrated in Figure S3 and our previous studies.^12,17^ Therefore, the piezoresponse observed in WSe_2_ is attributed to the flexoelectricity. Figure 2b shows a linear relation between the PFM amplitude and the AC voltage. Using the equation *d*_33,eff_ = *Δu*/*V*_AC_, where *Δu* is the tip deflection and *V*_AC_ is the applied AC voltage, we calculated the *d*_33,eff_ coefficient for the WSe_2_ nanoflake to be 0.29 pm/V based on the fitted curve. Even though the measured *d*_33,eff_ is close to the value previously reported for monolayer WSe_2_ (0.43 pm/V) on Au, it is much smaller than those of many vdW materials with out-of-plane mirror symmetry reported in the literature, such as 1.9 pm/V for multilayered WSe_2_ on heavily doped silicon, 2.0 pm/V for suspended 10-layer MoS_2_, and 9.4 pm/V for γ-InSe with a thickness of 22.1 nm.^14,18,19^ Thus, this suggests that the measured electromechanical response is likely affected by the supported substrates. Incorporating the coupling to the substrate into the theoretical modeling of PFM tests, Springolo et al. theoretically revealed that the response is highly sensitive to the substrate interaction strength.^20^ Indeed, in PFM experiments, the sharpness of the tip electrode generates inhomogeneous EF, which causes internal clamping in the materials. Such clamping effect can lead to a reduced mechanical deformation.^21,22^ When the materials are transferred onto the substrates, the interaction between them probably strengthens the clamping effect. In such cases, the supporting substrate usually results in a smaller *d*_33,eff_ coefficient.^23^

**Figure 2:**
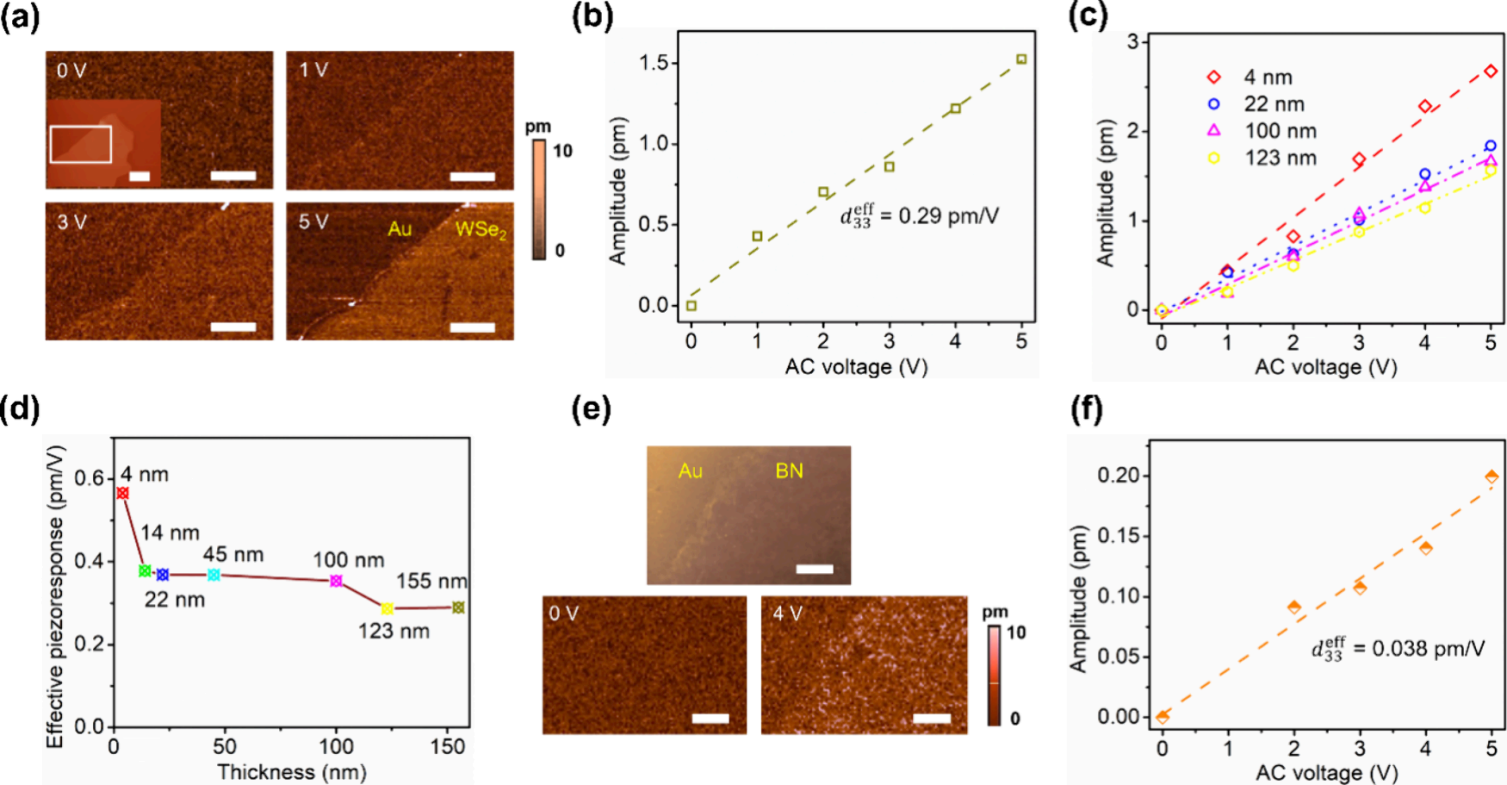
Out-of-plane electromechanical properties of WSe_2_ and monolayer h-BN. (a) PFM amplitude images of the framed region in the inset of panel a under AC voltages of 0, 1, 3, and 5 V. (b) PFM amplitude as a function of AC voltages of WSe_2_ in the inset of panel a. (c) PFM amplitudes as a function of AC voltages and (d) effective out-of-plane piezoelectric coefficients of WSe_2_ nanoflakes with varying thicknesses. (e) AFM topography image of monolayer h-BN on Au (top) and its corresponding PFM amplitude images under AC voltages of 0 V (bottom left) and 4 V (bottom right). (f) PFM amplitude as a function of AC voltage of monolayer h-BN. Scale bars of 5 μm in panels a and e and 10 μm in the inset of panel a.

We further measured the piezoresponse of WSe_2_ nanoflakes upon variation of their thickness, as shown in panels c and d of Figure 2. Our findings indicate that the *d*_33,eff_ coefficient decreases from 0.57 to 0.29 pm/V as the thickness of WSe_2_ increases, stabilizing at approximately 120 nm. The thickness dependence of *d*_33,eff_ can also be observed from the simulation results. As shown in Figure S4, the *d*_33,eff_ coefficient for monolayer WSe_2_ is 0.515 pm/V and decreases gradually with an increase in thickness. However, when the thickness increases to only 6.8 nm, *d*_33,eff_ reaches a stable value of approximately 0.12 pm/V. The slower decrease in *d*_33,eff_ with thickness or the larger *d*_33,eff_ coefficient measured in experiments is likely caused by the polar structure at the interface between WSe_2_ and Au substrates.^26–28^ The results from Kelvin probe force microscopy (KPFM) measurements (Figure S5) reveal that the contact potential difference (*V*_cpd_) of the thin WSe_2_ nanoflake on Au is smaller than that of the thicker WSe_2_ nanoflake on Au. This suggests that thinner WSe_2_ nanoflakes on Au exhibit a smaller work function. The change in the work function in TMDs upon variation of the thickness on a substrate may be attributed to factors such as the interlayer screening effect and the presence of adsorbed water and oxygen.^24,25^ The decrease in the work function of thinner WSe_2_ nanoflakes on Au indicates that more dipoles may form at the interface of the polar structure between WSe_2_ and the Au substrate. An increase in these dipoles can enhance the overall measured piezoresponse,^26,27^ resulting in a larger *d*_33,eff_ coefficient.

Previous research has demonstrated that the out-of-plane piezoelectric responses of low-dimensional vdW materials can be enhanced through heterostructure engineering. This enhancement is primarily attributed to structural symmetry breaking and/or the formation of interfacial polar structures.^12,16,27,29,30^ Due to its excellent chemical stability, mechanical flexibility, the absence of dangling bonds and charged impurities, and a large bandgap, h-BN is widely utilized as a dielectric substrate or protective layer in vdW electronics.^31,32^ Consequently, h-BN is a promising candidate for the fabrication of vdW heterostructures used in devices that exploit their electromechanical properties. While monolayer h-BN has weak effective out-of-plane piezoelectricity, the PFM amplitude of monolayer h-BN demonstrates a linear relationship with the AC voltage, showing a *d*_33,eff_ coefficient of 0.038 pm/V, as illustrated in panels e and f of Figure 2.

We further examined the out-of-plane electromechanical behaviors of the as-prepared WSe_2_/BN heterostructures. Figure 3a displays the PFM amplitude images of the WSe_2_/BN heterostructure depicted in Figure S1c. The contrast in the PFM image increases with applied AC voltage. Unlike the PFM images of WSe_2_, where the WSe_2_ regions appear brighter, the regions of WSe_2_/BN show darker contrast. To determine the true piezoresponse amplitude of WSe_2_/BN, we subtracted the background noise and plotted the result as a function of AC voltage.^12,13^ Notably, the PFM amplitude signal for WSe_2_/BN is negative, as shown in Figure 3b. The PFM amplitude for each WSe_2_/BN sample exhibits an inverse linear relationship with the AC voltage. The *d*_33,eff_ coefficient of WSe_2_/BN ranged from −0.12 to −0.19 pm/V, with these absolute values being smaller than those observed for WSe_2_.

**Figure 3:**
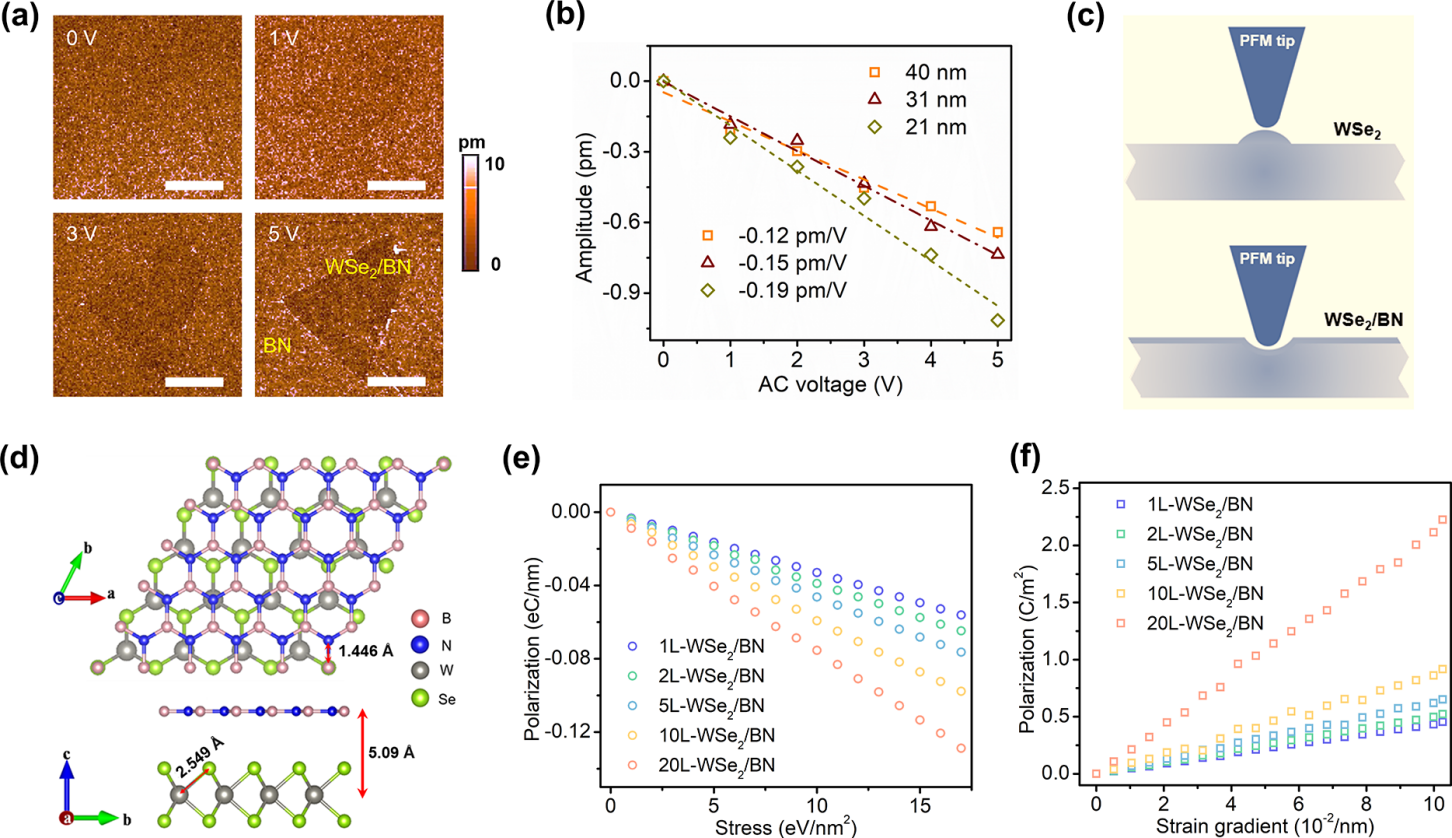
Out-of-plane electromechanical properties of WSe_2_/BN. (a) PFM amplitude images of WSe_2_/BN under AC voltages of 0, 1, 3, and 5 V. Scale bar of 10 μm. (b) PFM amplitudes as a function of AC voltages of WSe_2_/BN with varying thicknesses. (c) Schematics of volume expansion in WSe_2_ (top) and contraction in WSe_2_/BN (bottom) during PFM measurements. (d) Top (top) and side (bottom) views of simulated WSe_2_/BN. (e) Piezoelectric and (f) flexoelectric behaviors of simulated WSe_2_/BN.

Monolayer or odd-layered thin h-BN and 2H-TMDs belong to the *D*_3*h*_ point group, whose piezoelectric tensor can be expressed as

1 $$\left[\begin{array}{llllll} e_{11} & -e_{11} & 0 & 0 & 0 & 0\\ 0 & 0 & 0 & 0 & 0 & -e_{11}\\ 0 & 0 & 0 & 0 & 0 & 0 \end{array}\right]$$

where all of the out-of-plane piezoelectric coefficients are zero. Their out-of-plane piezoresponse has been experimental and theoretically verified to arise from flexoelectricity.^12–14,33^ In the case of WSe_2_, the measured *d*_33,eff_ coefficient was found to be positive, indicating a volume expansion, as illustrated in the top panel of Figure 3c. However, when a monolayer of h-BN was placed on top of WSe_2_, the measured *d*_33,eff_ became negative, indicating a volume contraction in the WSe_2_/BN system, as shown in the bottom panel of Figure 3c. Both pristine WSe_2_ and monolayer h-BN have positive *d*_33,eff_ coefficients due to flexoelectricity; therefore, the negative *d*_33,eff_ observed in WSe_2_/BN cannot be attributed to flexoelectricity.

To gain a deeper understanding of the NLP in WSe_2_/BN, we conducted simulations of bending deformations to examine the out-of-plane electromechanical properties of WSe_2_/BN. Detailed computational and simulation methods are provided in Supporting Note 1, Tables S1 and S2, and Figures S6 and S7. The simulated WSe_2_/BN heterostructures consist of a monolayer h-BN and WSe_2_ with different thicknesses (layer numbers). The unit cell for WSe_2_/BN was constructed by stacking a 5 × 5 supercell of single-layer BN on a 4 × 4 supercell of WSe_2_ (Supporting Note 1). Since the lattice constants of BN (*a* = 2.51 Å) and WSe_2_ (*a* = 3.18 Å) have a large discrepancy, the stacking of BN on WSe_2_ breaks the out-of-plane reflection symmetry at the interface. Such symmetry breaking in WSe_2_/BN can endow it with out-of-plane piezoelectricity. The typical optimized structure for WSe_2_/BN with a monolayer WSe_2_ (1L-WSe_2_/BN) is shown in Figure 3d. Although the thicknesses of the simulated heterostructures are smaller than those of the experimental samples, the simulation results can offer valuable insights into the origins of the NLP in WSe_2_/BN. The total out-of-plane electromechanical response in the simulated WSe_2_/BN is influenced by both flexoelectricity and piezoelectricity within the heterostructures. The out-of-plane polarization in relation to the stress and strain gradient is depicted in panels e and f, respectively, of Figure 3. As shown in Figure 3e, for all of the simulated WSe_2_/BN heterostructures, the piezoelectric polarization exhibits a negative linear correlation with stress. With an increase in thickness, the absolute values of the slopes increase, indicating negative intrinsic piezoelectric coefficients (*d*_int,33_) increasing from −0.327 pm/V for 1L-WSe_2_/BN to −0.744 pm/V for 20L-WSe_2_/BN (thickness of ∼13.93 nm), as shown in Figure S8. In contrast, for all of the simulated samples, the flexoelectric polarization increases linearly with the strain gradient (Figure 3f). From the slopes of the curves in Figure 3f, the effective piezoelectric coefficient (*d*_fle,33_) resulting from flexoelectricity is calculated to increase from 0.193 pm/V for 1L-WSe_2_/BN to 0.408 pm/V for 20L-WSe_2_/BN (Figure S8), suggesting that flexoelectricity has a dampening effect on the NLP of WSe_2_/BN. The overall *d*_33,eff_ coefficient of the simulated WSe_2_/BN heterostructures is shown in Figure S8, which is the sum of *d*_int,33_ and *d*_fle,33_. Although it seems that *d*_33,eff_ is slightly decreasing from −0.336 to −0.134 pm/V with an increase in thickness, it still has the same order of magnitude as that in our experimental findings.

NLP has been found in single-phase ferroelectric materials, including ABC ferroelectrics and 2D vdW layered ferroelectrics.^5,7,9,11^ In a phenomenological theory, the negative piezoelectricity of ferroelectric crystals can be understood as a result of spontaneous polarization-biased electrostriction.^9^ The atomic origins of NLP have been revealed through first-principles calculations that consider both “clamped-ion” and “internal-strain” terms. For instance, it was found that the “internal-strain” term is dramatically suppressed in low-dimensional ferroelectric CuInP_2_S_6_. As a consequence, the negative “clamped-ion” term dominates over the positive “internal-strain” term, leading to the occurrence of NLP.^11^ More recently, Chen et al. reported that even though low-dimensional heteroanionic vdW layered materials are not ferroelectric, the presence of preexisting out-of-plane polarization can still result in NLP in these 2D vdW materials. They conducted comprehensive simulations to explore a variety of heteroanionic vdW layered materials that exhibit NLP.^5^

Previous theoretical calculations have suggested that charge transfer can occur from WSe_2_ to h-BN, resulting in the formation of a built-in EF at the WSe_2_/BN junction.^34,35^ To investigate this built-in EF, we performed KPFM measurements on both WSe_2_ and WSe_2_/BN. Figures 4a displays the KPFM results for the WSe_2_ nanoflake (top panel) and WSe_2_/BN (bottom panel). Both samples have similar thicknesses, as shown in Figure S9. The statistical distributions of *V*_cpd_ (Figure 4b) indicate that the *V*_cpd_ of WSe_2_/BN is 162.7 mV lower than that of WSe_2_. This suggests that the work function for WSe_2_/BN is higher, which may imply the presence of a depletion region in WSe_2_/BN.

**Figure 4:**
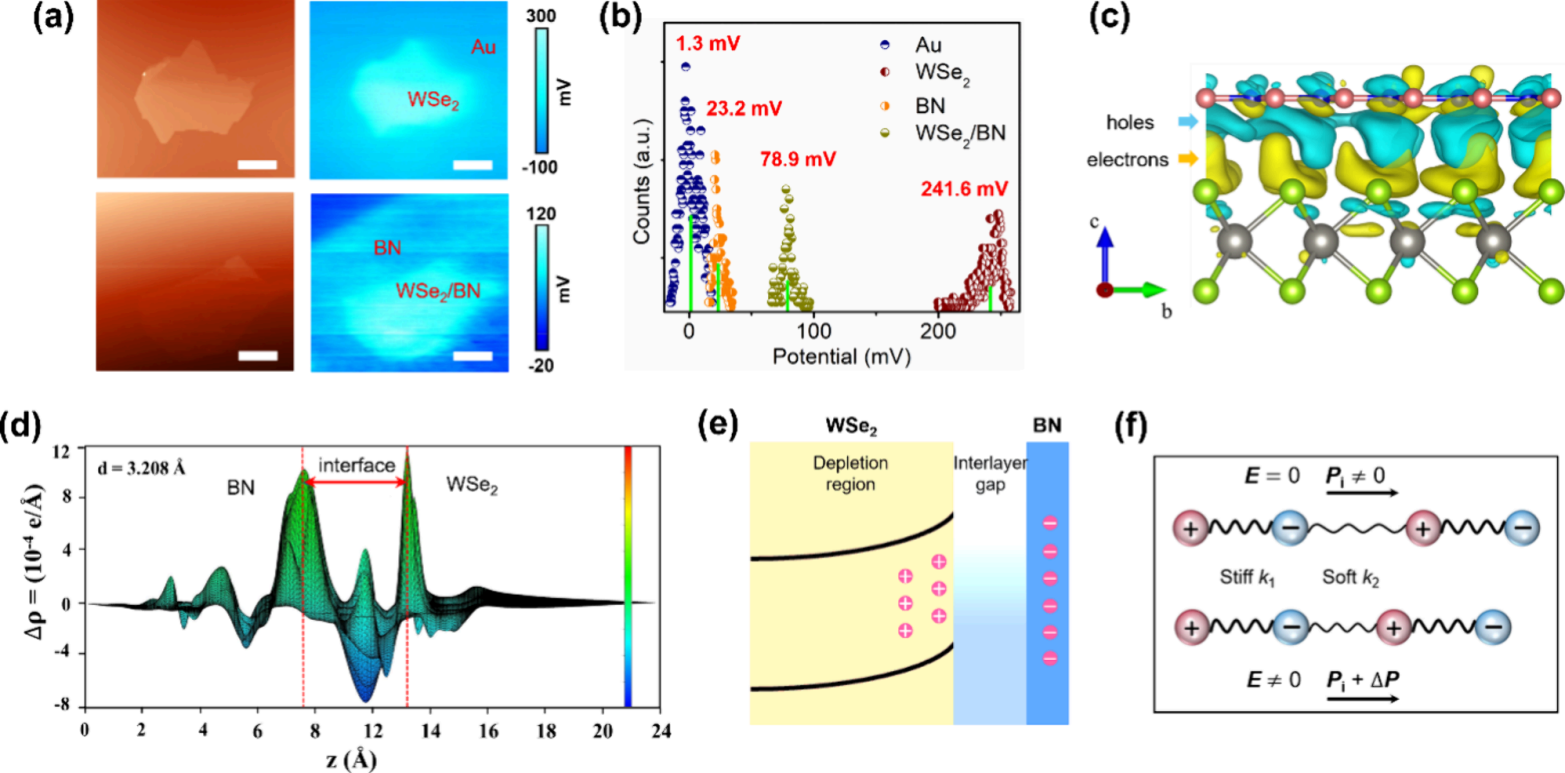
Mechanisms of NLP in WSe_2_/BN. (a) AFM topography image (top left) of a WSe_2_ nanoflake and its corresponding KPFM image (top right) and AFM topography image of a WSe_2_/BN heterostructure (bottom left) and its corresponding KPFM image (bottom right). The scale bar is 10 μm. (b) Statistic distributions of the surface potential difference for the Au substrate, monolayer h-BN, WSe_2_ nanoflake, and WSe_2_/BN. (c) Side view of the charge density difference (*Δρ*) for Bi_2_Se_3_/BN. The cyan and yellow colors indicate the depletion and accumulation of electrons, respectively. The isovalues are 10^–4^ e/Å^3^. (d) Planar-averaged electron density difference of Bi_2_Se_3_/BN. The positive and negative values of *Δρ* indicate the accumulation and depletion of electrons, respectively. (e) Schematic of the built-in EF in WSe_2_/BN. (f) Schematic of the negative piezoelectricity of the depletion region in vdW heterostructures explained by the ball-and-spring model.

We calculated the charge density difference (*Δρ*) of the heterostructures, which is described as^35^

2 $$\Delta \rho =\rho_{het}-\rho_{BN}-\rho_{s}$$

where *ρ*_het_, *ρ*_BN_, and *ρ*_s_ represent the charge densities of the heterostructures, the monolayer h-BN, and the pristine WSe_2_ or Bi_2_Se_3_ (shown below), respectively. As illustrated in Figure 4c (with a top view shown in Figure S10), the spatial charge accumulation and loss across the heterostructure are labeled as yellow and cyan, respectively. Due to the charge redistribution, electrons predominately accumulated near WSe_2_ while being depleted near BN.^35^ Clearly, some electrons were transferred from WSe_2_ to BN, which is also evident from the planar-averaged charge density difference along the stacking direction (*z*), as shown in Figure 4d. The flow of charge from WSe_2_ to BN induces band bending and creates a space charge region in WSe_2_, which generates a depletion region, as depicted in the schematic of the WSe_2_/BN heterojunction in Figure 4e, resembling a Schottky junction. Previous studies found that the depletion region in heterojunctions can lead to interface piezoelectricity, resulting from the combination of the built-in EF and the electrostriction effect.^27^ Similarly, WSe_2_/BN can also exhibit an interface piezoelectricity in its depletion region. Moreover, the built-in polarization that exists in the vdW WSe_2_/BN heterostructure is similar to the out-of-plane polarization in heteroanionic vdW layered materials, which contributes to NLP.^5^ Therefore, the negative value of *d*_33,eff_ for WSe_2_/BN is most likely due to the negative electrostriction effect present in the interface depletion region.

Previous studies have employed a ball-and-spring model to qualitatively explain the microscopic origin of negative piezoelectricity in vdW ferroelectrics and heteroanionic vdW layered materials that exhibit intrinsic out-of-plane polarization.^5,9^ The WSe_2_/BN heterostructures can be understood as a stack of single layers held together by weak vdW forces. In this model, the ions and bonds are simplified as balls and springs, respectively, with each layer acting as an electric dipole. The strong intralayer bonds and the weak interlayer bonds are represented by elastic compliances, *k*_1_ and *k*_2_ (*k*_1_ ≫ *k*_2_), respectively, as illustrated in Figure 4f. The built-in EF varies with the applied external EF, which narrows the vdW gaps in the depletion region. Meanwhile, the monolayer thickness remains almost inert due to the softness of the interlayer gaps. Consequently, the application of AC voltages induces NLP. In contrast, nonlayered semiconductors with strong covalent/ionic bonds, such as niobium-doped SrTiO_3_ (Nb:STO), lack such weak interlayer bonds, making it easier for expansion to occur in the depletion region through the electrostriction effect when an electric filed is applied.^27^

It is important to note that without the built-in polarization and the resulting negative electrostriction effect, NLP may not be induced in heterostructures comprised of nonpiezoelectric vdW materials. For example, we previously fabricated MoS_2_/BN heterostructures by stacking monolayer h-BN on 2H-MoS_2_ nanoflakes. In this case, since there was no charge transfer between MoS_2_ and h-BN, a built-in EF was absent. However, the broken structural symmetry and the interface polar structure caused by the surface defects or lattice strain in the MoS_2_/BN heterostructures led to enhanced positive longitudinal piezoresponses.^12^ To further understand NLP in vdW heterostructures, we examined the out-of-plane electromechanical properties of Bi_2_Se_3_/BN, where the Bi_2_Se_3_ nanoflakes also have no out-of-plane piezoelectricity.^17^ Figure 5b displays the PFM amplitude images of the Bi_2_Se_3_/BN heterostructure (with the thickness shown in Figure S11) shown in Figure 5a as the AC voltage increases. In comparison to BN, the Bi_2_Se_3_/BN region shows a darker image contrast, indicating a weaker piezoresponse or NLP. As shown in Figure 5c, the PFM amplitude of Bi_2_Se_3_/BN increases linearly with the AC voltage. Different from WSe_2_/BN, the overall effective piezoelectricity of Bi_2_Se_3_/BN does not show a negative sign but reduced piezoresponses. The *d*_33,eff_ coefficient for the latter is calculated to be only 0.024 pm/V, approximately 1 order of magnitude smaller than that of the Bi_2_Se_3_ nanoflakes reported in our previous work.^17^ Table S3 shows a comparison of *d*_33,eff_ for pristine MoS_2_, WSe_2_, Bi_2_Se_3_, and the heterostructures formed by stacking monolayer BN on them, where only WSe_2_/BN exhibits a negative *d*_33,eff_ coefficient.

**Figure 5:**
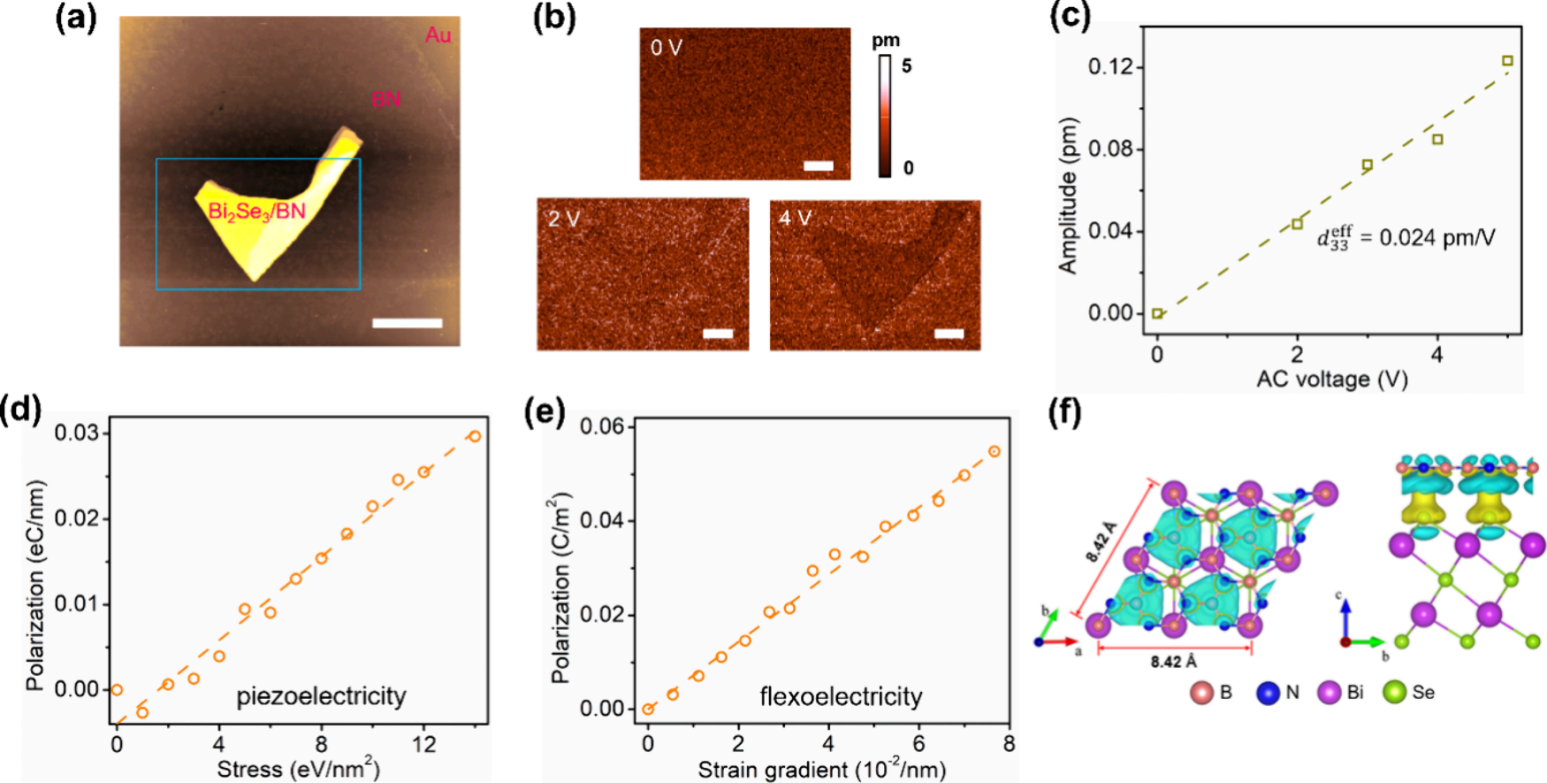
Out-of-plane electromechanical performances of Bi_2_Se_3_/BN. (a) AFM topography image of Bi_2_Se_3_/BN. (b) PFM amplitude images of the Bi_2_Se_3_/BN heterostructure under AC voltages of 0, 2, and 4 V. (c) PFM amplitudes as a function of AC voltages of the Bi_2_Se_3_/BN heterostructure. (d) Piezoelectric behavior and (e) flexoelectric behavior of the simulated Bi_2_Se_3_/BN. (f) Top (left) and side (right) views of the charge density difference of simulated Bi_2_Se_3_/BN. Scale bars of 20 μm in panel a and 2 μm in panel b.

The simulation results presented in panels d and e of Figure 5 indicate that both piezoelectric polarization and flexoelectric polarization in Bi_2_Se_3_/BN increase linearly. The overall *d*_33,eff_ for Bi_2_Se_3_/BN is calculated to be only approximately 0.011 pm/V, which is on the same order of magnitude as our experimental findings. The “suppressed” flexoelectricity in BN-capped Bi_2_Se_3_ is likely attributed to the increased stiffness of the Bi_2_Se_3_/BN structure compared to Bi_2_Se_3_.^36^ The charge density difference image (Figure 5f) of Bi_2_Se_3_/BN reveals that there is also an obvious charge exchange between Bi_2_Se_3_ and BN, indicating that a built-in EF might be formed in the Bi_2_Se_3_/BN heterostructure. The charge transfer is confirmed by the KPFM results shown in Figure S12, where an increase in the work function of Bi_2_Se_3_/BN compared to that of Bi_2_Se_3_ can be found (with thicknesses shown in Figure S13). Based on phenomenological theories, we assume that the overall *d*_33,eff_ coefficient for the heterostructure could be written as *d*_33,eff_ = *d*_33,bi_ + *d*_33,fle_ + *d*_33,mL_, where *d*_33,bi_ describes the interface piezoelectricity due to the built-in EF, *d*_33,fle_ reflects the effective piezoelectricity stemming from flexoelectricity, and *d*_33,mL_ represents the piezoelectricity due the polar structure in WSe_2_ or Bi_2_Se_3_ layers caused by the symmetry breaking, the lattice strain, and the charge transfer (see Supporting Note 3).^16,27^ Due to the “soft” nature of the interlayer gaps in vdW heterostructures, *d*_33,bi_ is likely to be negative. When the absolute value of *d*_33,bi_ is smaller than the sum of *d*_33,fle_ and *d*_33,mL_, such as the case for Bi_2_Se_3_/BN, only a weak positive piezoresponse instead of NLP is observed. Therefore, it is expected that a stronger built-in EF would cause a larger negative *d*_33,eff_.

In summary, we have demonstrated negative interface piezoelectric effects in nonpiezoelectric vdW layered materials that are capped with a monolayer of h-BN. The observed NLP in the prepared heterostructures is attributed to the weak interlayer bonds and the built-in EF at the heterojunction. There is a competitive relationship between the interface NLP and the flexoelectricity of the heterostructures. The *d*_33,eff_ coefficient of the heterostructures becomes negative when the interface NLP is stronger than the flexoelectric effect. Although the NLP observed for WSe_2_/BN is not large, our findings enhance the understanding of NLP in vdW heterostructures and provide a method for controlling the sign of the out-of-plane electromechanical response of low-dimensional vdW materials. We anticipate that our work will expand the potential applications of nonpiezoelectric vdW materials in devices that utilize electromechanical properties. Furthermore, our findings suggest that the coupling effects between the heterojunctions and electromechanical properties in electronics based on vdW heterostructures should be carefully considered, particularly in piezoelectronics.

## Methods

*Sample Fabrication*. High-quality (purity of >99.995%) hexagonal WSe_2_ and Bi_2_Se_3_ crystals were commercially available from HQ Graphene Inc. Monolayer h-BN films grown on Si/SiO_2_ wafers were purchased from Grolltex. Gold-coated Si substrates (1 cm × 1 cm) were prepared by using a gold sputter system (JEOL JFC-1300). A typical mechanical exfoliation method using Scotch tape was used to obtain WSe_2_ and Bi_2_Se_3_ nanoflakes. Homemade poly­(dimethylsiloxane) (PDMS) stamps were used to transfer the WSe_2_ or Bi_2_Se_3_ nanoflakes onto the gold-coated Si substrates.

A wet transfer method was adopted to fabricate heterostructures of WSe_2_/BN and Bi_2_Se_3_/BN.^12^ Briefly, a poly­(methyl methacrylate) (PMMA) layer was spin-coated onto monolayer h-BN grown on Si/SiO_2_ substrates at a speed of 2500 rpm. The monolayer h-BN/PMMA films were detached from the Si/SiO_2_ substrates by etching in a 1 M KOH solution at 70 °C for 3 h, followed by thorough rinsing in deionized water. The floating h-BN/PMMA film was then transferred onto a gold-coated Si substate with WSe_2_ or Bi_2_Se_3_ nanoflakes. This was followed by immersion in acetone for 3 h and in 2-propanol for 20 min.

*Characterization*. An optical microscope (Leica DM1750 M) and an AFM instrument (NTEGRA Prima, NT-MDT Co.) were used to characterize the surface morphology. Electromechanical effects and surface potential were studied using PFM and KPFM on the NTEGRA Prima scanning probe microscope under ambient conditions. A Cr/Au-coated silicon probe (HQ:NSC18/Cr-Au, μMasch) was used with a spring constant of ∼2.8 N/m and a free resonance frequency of ∼75 kHz.

*Computational Details*. See Supporting Note 1.

## Supplementary Material



## Notes

Morphological characterization, configuration of PFM tests and determination of AC voltage frequency, PFM results of WSe_2_ under DC voltages, effective out-of-plane piezoelectric coefficients, KPFM results of WSe_2_, computational details, simulated bending deformation, out-of-plane electromechanical performance of simulated WSe_2_/BN, line profiles of the height for WSe_2_ and WSe_2_/BN, charge density difference of WSe_2_/BN, line profile of height for WSe_2_/BN, KPFM results of Bi_2_Se_3_ and Bi_2_Se_3_/BN, line profile of Bi_2_Se_3_ and Bi_2_Se_3_/BN, summary of effective out-of-plane piezoelectric coefficients, and phenomenological theory of *d*_33,eff_ (PDF)
