# Supplementary material for: Negative Out-of-Plane Electromechanical Response in Nonpiezoelectric van der Waals Layered Materials Encapsulated by Monolayer Boron Nitride
Source: J Phys Chem Lett. 2025 Oct 9;16(42):10848–55. doi: 10.1021/acs.jpclett.5c02427 (PMC12557388; doi:10.1021/acs.jpclett.5c02427)
Supplement: Supplementary file 1 [file jz5c02427_si_001.pdf]

Supporting Information

Negative out-of-plane electromechanical response in non-piezoelectric van der Waals layered materials encapsulated by monolayer boron nitride

Qiong Liu,[1,2,6] Vijay Kumar Choyal,[1] Han Hu,[1] Timon Rabczuk,[3] Xiaoning Jiang,[4] and Xiaoying Zhuang,[1,5,6]*

[1]Institute of Photonics (IOP), Faculty of Mathematics and Physics, Leibniz University Hannover, Hannover 30167, Germany

[2]Laboratory of Nano and Quantum Engineering (LNQE), Leibniz University Hannover, Hannover 30167, Germany

[3]Institute of Structural Mechanics, Bauhaus University, Weimar 99423, Germany

[4]Department of Mechanical and Aerospace Engineering, North Carolina State University, Raleigh, NC 27695-7910, United States of America

[5]Department of Geotechnical Engineering, College of Civil Engineering, Tongji University, Shanghai 200092, China

[6]Cluster of Excellence PhoenixD (Photonics, Optics and Engineering – Innovation Across Disciplines), Hannover 30167, Germany

Email: zhuang@iop.uni-hannover.de

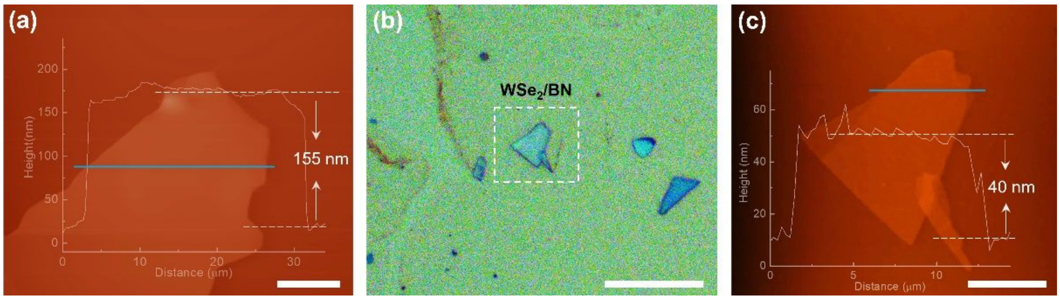

Figure S1. Morphological characterization. (a) AFM topography image of a WSe_2 nanoflake. Inset is the height profile along the solid line. (b) Optical microscopy image of Bi_2Se_3/BN heterostructures. (c) AFM topography image of the Bi_2Se_3/BN heterostructure in the framed region in (b). Inset is the height profile along the solid line. Scale bar, 10 μm in (a) and (c), 50 μm in (b).

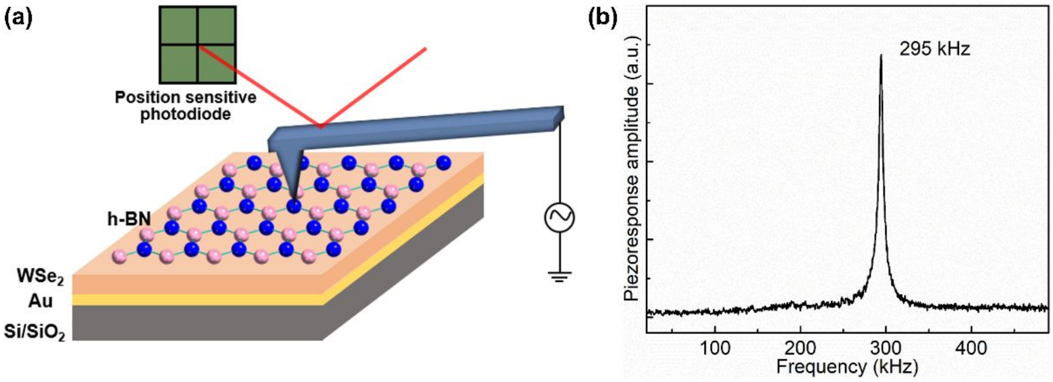

Figure S2. Illustration of PFM measurements. (a) Schematic of PFM measurements. (b) Determination of the frequency of the AC voltages for the PFM measurements. The contact resonance frequency of the AFM cantilever is approximately 295 kHz.

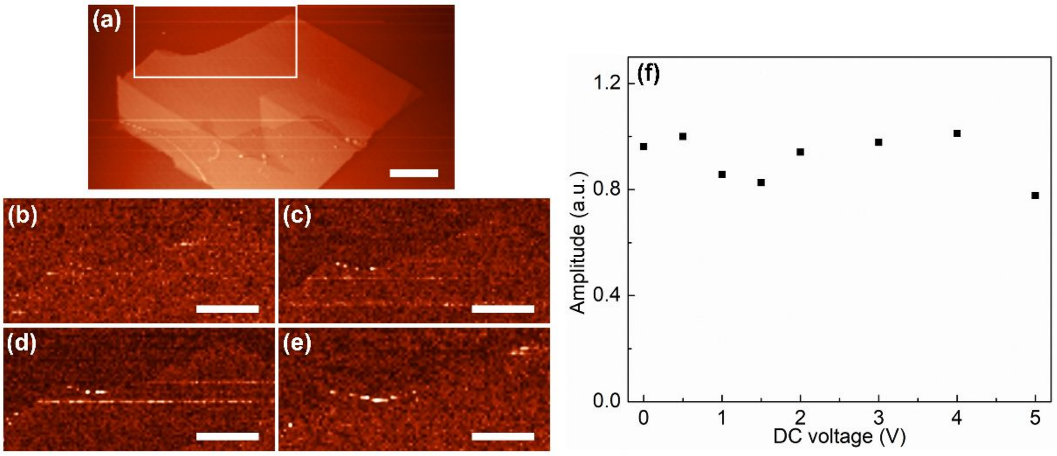

Figure S3. PFM results of a single WSe_2 nanoflake under an AC voltage of 4 V with different DC voltages. (a) AFM topography image of the WSe_2 nanoflake. (b-e) PFM amplitude images measured under DC voltages 0, 1, 2, and 5 V, respectively. (f) PFM amplitude versus DC voltage. Scale bar, 10 μm.

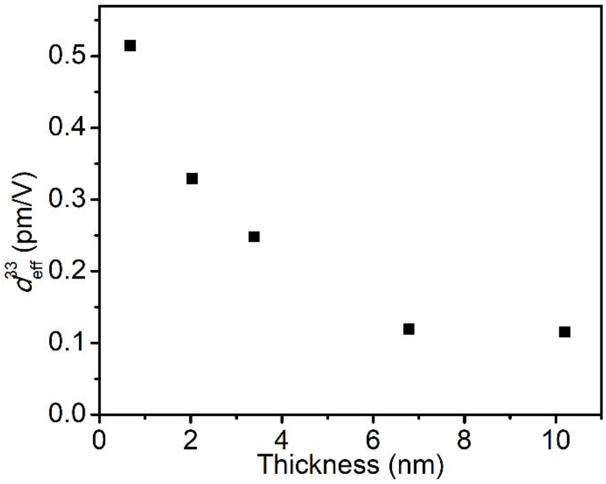

Figure S4. Effective out-of-plane piezoelectric coefficient as a function of the thickness for simulated WSe_2.

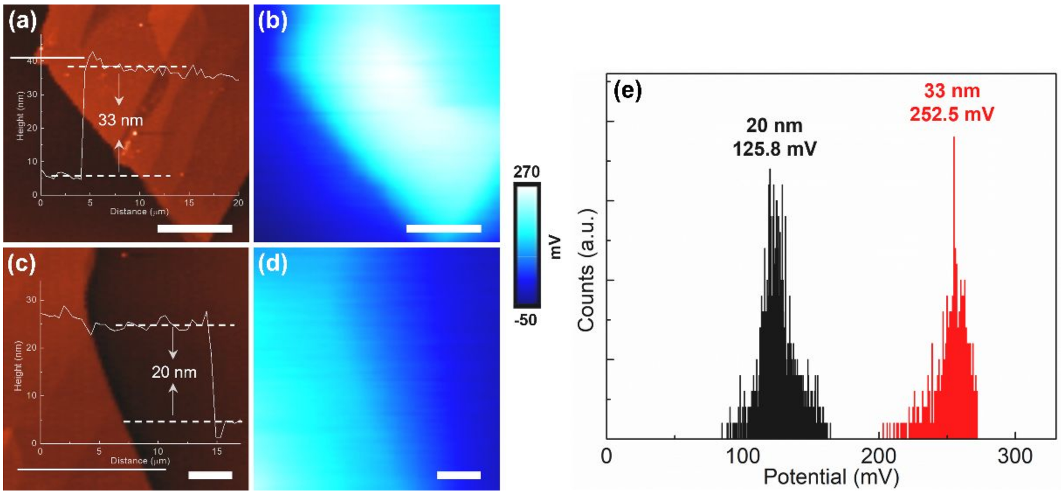

Figure S5. KPFM results of WSe_2. (a) AFM topography image of a WSe_2 nanoflake with a thickness of 33 nm (inset: line profile of height along the solid line), and (b) its corresponding KPFM image. (c) AFM topography image of a WSe_2 nanoflake with a thickness of 20 nm (inset: line profile of height along the solid line), and (d) its corresponding KPFM image. (e) Statistic distributions of the contact potential difference for the WSe_2 nanoflakes. Scale bar, 20 μm in (a) and (b), 5 μm in (c) and (d).

Supporting Note 1 Computational details

Density functional theory (DFT) simulations were conducted using the Vienna Ab-initio Simulation Package (VASP).[1,2] The generalized-gradient approximation (GGA) with the Perdew–Burke–Ernzerh (PBE) exchange correction functional was adopted with a cut-off energy of 500 eV for the plane waves. Energy minimization was achieved with the conjugate gradient approaching the convergence criteria of 10^{-5} eV for the electronic self-consistent loop. The Ab-initio molecular dynamics (AIMD) simulations were performed under Nosé thermostat with time step 1 fs using 2 x 2 x1 k-points grids.[3] The AIMD simulations were also carried out at 200 and 1500 K for WSe_2/BN, and 200 and 1000K for Bi_2Se_3/BN, respectively, with each for 1000-time steps, and half of the full trajectories selected to create the training sets. The training sets were prepared by conducting AIMD simulations over a 4 x 4 x 1 supercell of WSe_2 and a single-layer 5 x 5 x 1 supercell of BN with 2 x 2 x 1 k-points grids. More simulated WSe_2/BN heterostructures were fabricated by varying the layer numbers of WSe_2. For Bi_2Se_3/BN,

it is a 3 x 3 x 1 supercell of Bi_2Se_3 and a single-layer 2 x 2 x 1 supercell of BN with 2 x 2 x 1 k-points grids. For instance, the resulting WSe_2/BN heterostructure with a single-layer WSe_2 has a total of 106 atoms and 0.33% lattice mismatch, while Bi_2Se_3/BN with a single layer Bi_2Se_3 has a total of 107 atoms and approximately 0.52% lattice mismatch. Although the strain in the simulated Bi_2Se_3/BN here is not as small as WSe_2/BN, it is much smaller than that in the synthesized Bi_2Se_3/BN heterostructure.[4] The moment tensor potential (MTP) parameters for every structure were then passively fitted using the methodology adopted in our earlier studies.[5,6]

In the present study, we also compared the energies (eV/atom) and forces (eV/Å) obtained from AIMD and MLIP for the Bi_2Se_3/BN heterostructure. The precision and accuracy of newly developed MLIP were quantitatively assessed by comparing its predictions with those from DFT-based AIMD simulations. The trained MLIP of Bi_2Se_3/BN demonstrated outstanding predictive capability, as illustrated in Figure S6. Moreover, the accuracy of MLIP depends on minimizing the root mean squared errors (RMSEs) against target metrics (total energies (meV/atom) and atomic forces (meV/Å) and optimizing key hyperparameters. Figure S6a shows a comparison between the energies and forces obtained from AIMD simulations and their corresponding values fitted by MLIP for Bi_2Se_3/BN heterostructure in the training and test datasets. The RMSE was found to be 2.69×10^{-4} (eV/atom) for the training data and 4.464×10^{-4} (eV/atom) for the test data. Similarly, the forces (f_x, f_y, and f_z) obtained from both training (test) datasets are 0.0550 (0.0520), 0.0378 (0.0371), and 0.0741 (0.0738), respectively. We can see that the RMSEs from the training and test datasets are similar. These low error margins suggest high precision during the potential training phase in Bi_2Se_3/BN heterostructure. The trend lines and calculated absolute differences for energies and forces highlight the exceptional predictive ability of the developed MLIPs. Hence, the outcomes indicate that the trained MLIP is highly accurate and suitable for conducting atomistic simulations on Bi_2Se_3/BN heterostructure.

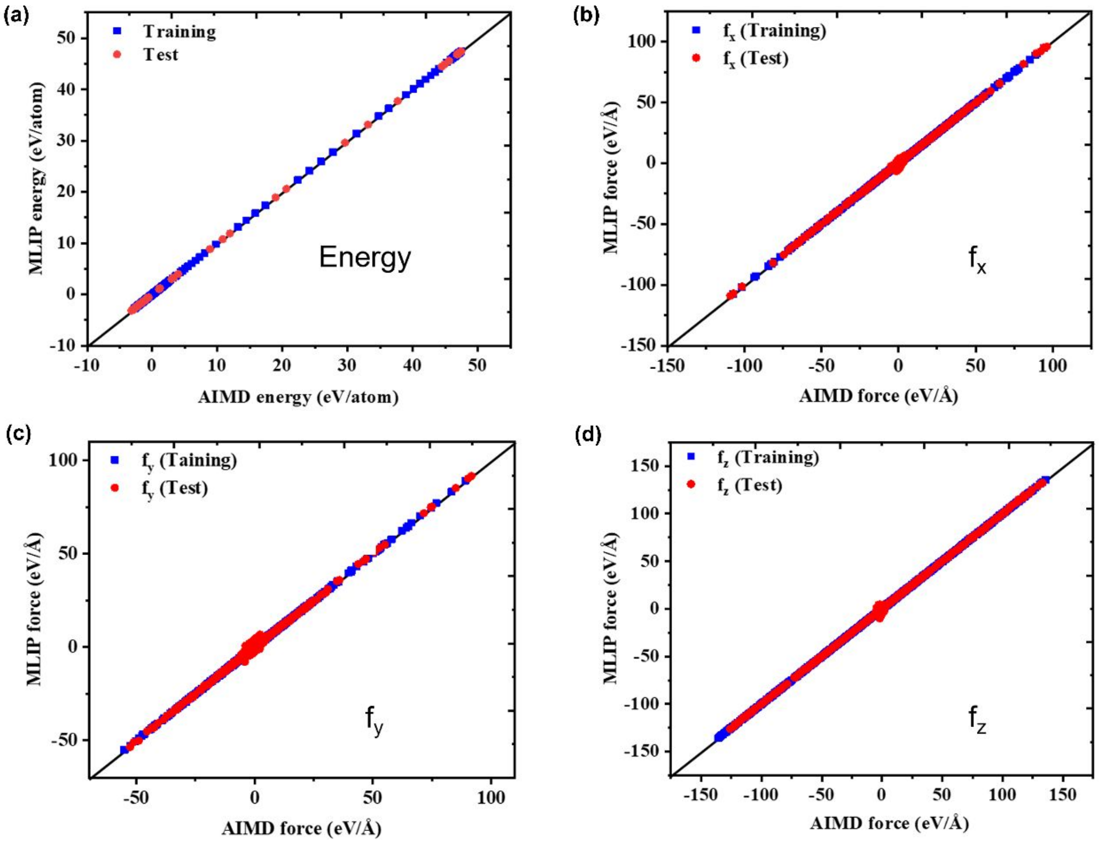

Figure S6. Comparison of the energies (eV/atom) and forces (eV/Å) derived from AIMD simulation and MLIP for training and test datasets of Bi_2Se_3/BN heterostructure, with training and test data points represented in blue and red color and the identity line represented in black color.

Table S1. Lattice thickness L (Å), inter-layer energy Φ_0 (eV) and distance h_0 (Å) from the derived interatomic potential for the heterogeneous structures; the atomic polarizability (α_{DFT}) calculated from DFT and that (α_{CD}) derived from CD model respectively; the elastic modulus (Y) obtained from MD simulations.

No.	Configurations	L (Å)	Φ_0 (eV)	h_0 (Å)	α_{DFT} (Å^3)	α_{CD} (Å^3)	Y (GPa)
1	WSe_2/BN	9.8	-0.0504	3.250	438.18	440.29	455
2	Bi_2Se_3/BN	12.9	-0.0834	3.428	324.60	320.41	136

We used classical MD simulations performed by Large-scale Atomic/Molecular Massively Parallel Simulator (LAMMPS) to calculate the deformation-induced electric polarization for pristine WSe_2 and different heterostructures.[7] We added the charge-dipole (CD) model to the short-range MTP potential derived from the AIMD simulations. According to the CD model, each atom carries a charge q and a dipole moment $\mathbf{p}$. This model depends on the parameter R, which represents the Gaussian distributed charge width for each atom type. Parameter R is related to the total polarizability. We tuned R to match the atomic polarizability (α_{DFT}) estimated from DFT calculation using GAUSSIAN software to that (α_{CD}) obtained from the CD model (see Table 1).[8] Next, we calculate the long-range vdW inter-layer interaction parameters for WSe_2/BN and Bi_2Se_3/BN heterostructures. The sizes of the unit cells for WSe_2/BN and Bi_2Se_3/BN along the z-direction were set to 50 Å and 20 Å, respectively, to avoid unnecessary interaction. We tuned the inter-layer distance h (Å) between the BN monolayer and WSe_2 or Bi_2Se_3 in the unit cell and performed electronic self-consistent calculations taking into account the vdW dispersion correction using the DFT-D3 method of Grimme.[9] We recorded the total energies of the WSe_2/BN and Bi_2Se_3/BN systems as a function of inter-layer distances. Then, we performed classical single-step MD simulations with no velocity involvement over a simulation cell area (5 nm × 4 nm) for WSe_2/BN and (3 nm × 4 nm) for Bi_2Se_3/BN with in-plane (x and y) periodic boundary conditions. The generated MTP parameters (ε) and Lennard-Jones parameters (σ) define the pair interaction potential of the heterostructures (ε and σ listed in Table S2). The defined inter-layer energy Φ_0 and distance h_0 from MD simulations are listed in Table S1. The calculation procedure for deriving the long-range vdW inter-layer interactions has already been described in previous studies.[10,11]

Table S2. The generated MTP parameters (ε) and Lennard-Jones potential parameters (σ) for WSe_2/BN and Bi_2Se_3/BN used in MD simulations.

No.	Interaction between different atoms	$\varepsilon = \sqrt{\varepsilon_1 \varepsilon_2}$ (eV)	$\sigma = \frac{\sigma_1+\sigma_2}{2}$ (Å)
1	W–B	0.0045370	3.115
2	W–N	0.0100362	3.538
3	Se–B	0.0038556	2.752
4	Se–N	0.0085290	3.274
5	Bi–B	0.0245041	3.418
6	Bi–N	0.0456917	3.942
7	Se–B	0.0348103	3.517
8	Se–N	0.0179035	3.871

We conducted in-plane tensile deformation simulations to obtain the Young's modulus, where we used a fixed size of 100 Å × 100 Å for unit cells. The linear displacement field is defined as $u_y = Ky$ along the *y*-axis, where *K* represents the atomic strain in a given direction, and *y* represents the atomic coordinates in the *y*-direction. To perform bending deformation, we considered bending displacement field $u_z = \frac{1}{2}Ny^2$, where *N* represents the strain gradient of the bending plane. Then, the edge region atoms were fixed while the interior atoms were relaxed to energy minimizing positions using the constant temperature simulations (NVT ensemble). Then, we calculated the point charges and dipole moments for each atom using the CD model. The total polarization of the systems was calculated as

$$\mathbf{P} = \frac{\sum_{i=1}^{n} \mathbf{P}_i}{V} \quad (1)$$

where *V* is the volume of the unit cell, an n is the total number of atoms. The total polarization is derived from both piezoelectricity and flexoelectricity, which can be described as

$$\mathbf{P} = d_{\alpha\beta\gamma}\varepsilon_{\beta\gamma} + \mu_{\alpha\beta\gamma\delta}\frac{\delta\varepsilon_{\beta\gamma}}{\delta r_\delta} \quad (2)$$

where $d_{\alpha\beta\gamma}$ and $\mu_{\alpha\beta\gamma\delta}$ represent the piezoelectric and flexoelectric coefficients,

respectively; $\varepsilon_{\beta\gamma}$ and $\frac{\delta\varepsilon_{\beta\gamma}}{\delta r_{\delta}}$ are the strain and strain gradient of unit cell, respectively.

The initial simulations were performed to benchmark the derived MTP's parameters. The in-plane elastic modulus (Y) under tensile deformation was estimated from the slope of stress-strain curve (up to 1%) (not shown here). The elastic moduli of WSe_2/BN and Bi_2Se_3/BN were calculated to be 455 and 135 GPa, respectively (Table S1). Note that we avoided using the thickness in calculating stress and polarization. The compressive loading simulations were performed to investigate the bending stiffness (D_{b} (eV)) and flexoelectric coefficient (μ_{zyzy}), as shown in a typical case for WSe_2/BN (Figure S7). The procedure in the previous study was adopted for calculating the bending stiffness from the linear relation of the bending energy and the square of curvature (κ^2).[11] The flexoelectric coefficient was obtained from the linear variation of the out-of-plane polarization (P_{z} (C/m^2)) and strain gradient (Å^{-1}). The effective piezoelectric coefficient (d_{zzz}) derived from flexoelectricity can be obtained under the assumption of small length scales and linear electric fields (EFs) using the equation $\mu_{zyzy} = d_{\mathrm{zzz}} Y L/2$.[12]

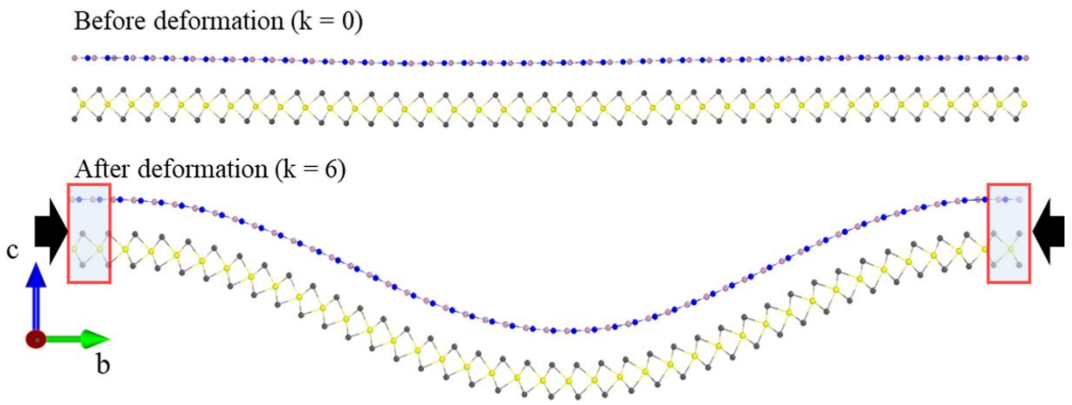

Figure S7. Bending deformation simulation of WSe_2/BN heterostructures.

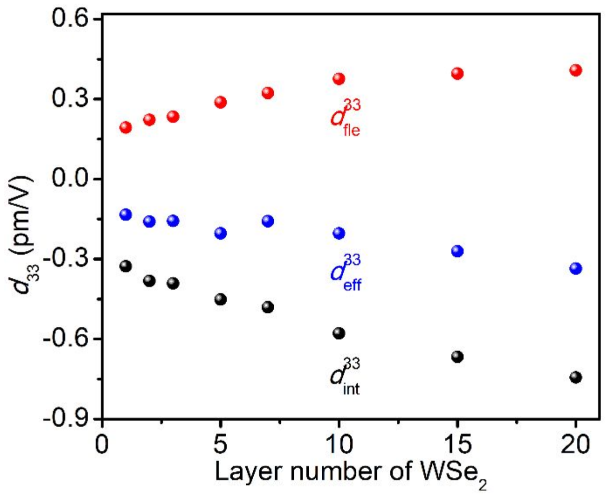

Figure S8. *d*fle 33, *d*int 33and *d*eff 33 as a function of the thickness of WSe_2 for the simulated WSe_2/BN, respectively.

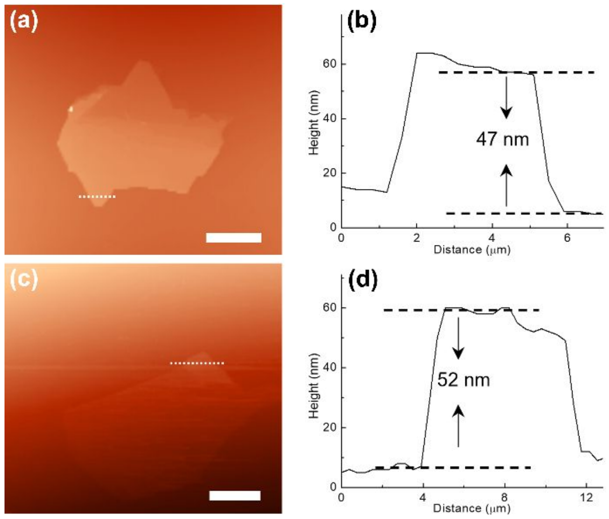

Figure S9. Illustration of thicknesses for WSe_2 and WSe_2/BN. (a) AFM topography image of a WSe_2 nanoflake and (b) the line profile of height along the dashed line in (a). (c) AFM topography image of a Bi_2Se_3/BN heterostructure and (d) the line profile of height along the dashed line in (c). Scale bar, 5 μm in (a), 10 μm in (c).

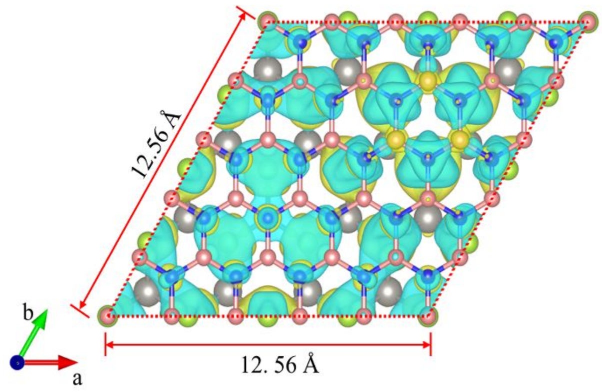

Figure S10. Top view of the charge density difference of WSe_2/BN. The cyan and yellow color describes the iso-surfaces corresponding to the accumulation and depletion of electrons. The Iso-values are 10^{-4} e/Å^3.

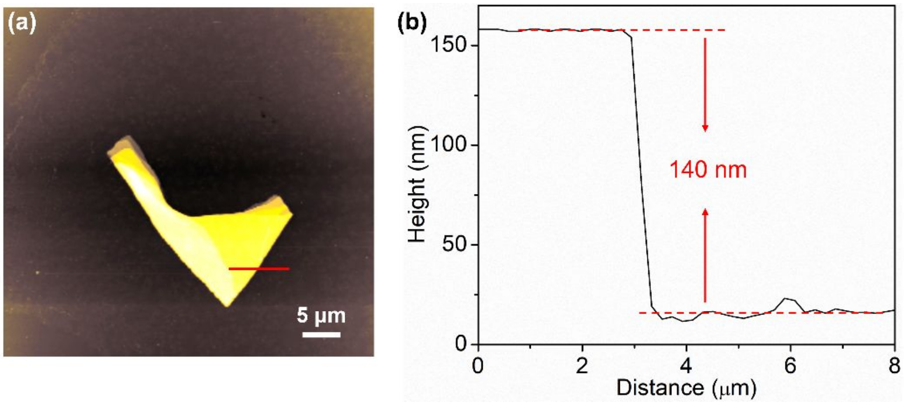

Figure S11. Illustration of the thickness for Bi_2Se_3/BN shown in Figure 5a. (a) AFM topography image and (b) the line profile of height along the dashed line in (a).

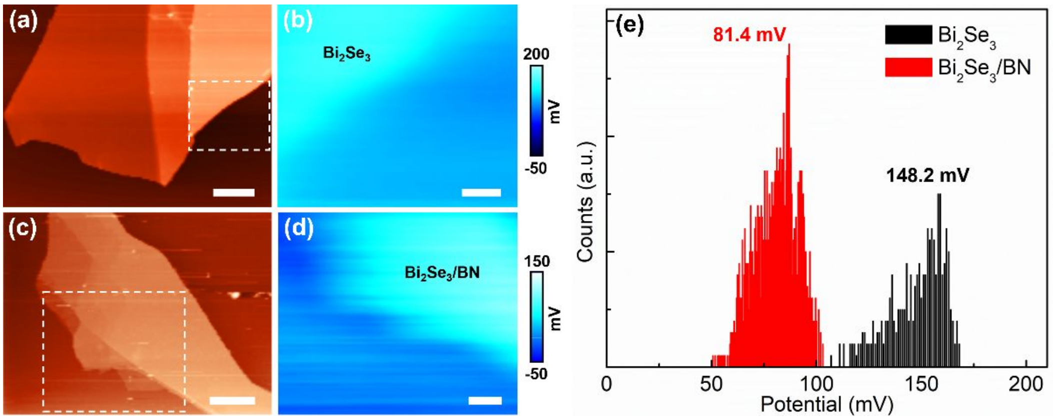

Figure S12. KPFM results of Bi_2Se_3 and Bi_2Se_3/BN. (a) AFM topography image of a Bi_2Se_3 nanoflake and (b) its corresponding KPFM image of the framed region. (c) AFM topography image and (d) its corresponding KPFM image of the framed region. (e) Statistic distributions of the surface potential difference for Bi_2Se_3 in (a) and Bi_2Se_3/BN in (c). Scale bar, 5 μm in (a) and (d), and (i), 2 μm in (b), 10 in (c).

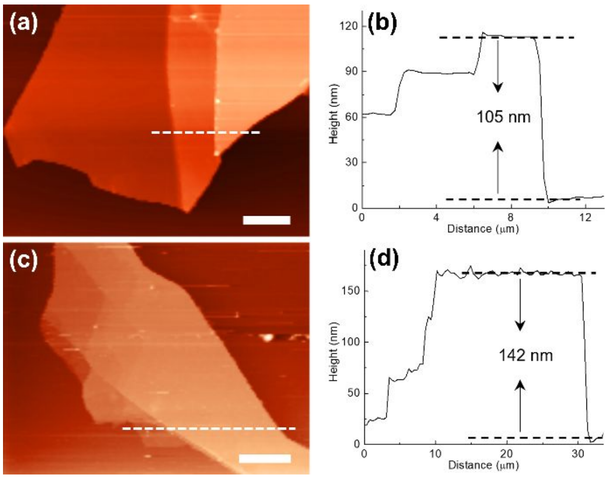

Figure S13. Illustration of thicknesses for Bi_2Se_3 and Bi_2Se_3/BN in Figure S12. (a) AFM topography image of the Bi_2Se_3 nanoflake and (b) the line profile of height along the dashed line in (a). (c) AFM topography image of the Bi_2Se_3/BN heterostructure and (d) the line profile of height along the dashed line in (c). Scale bar, 5 μm in (a), 10 μm in (c).

Table S3. Comparison of $d_{33}^{\,\text{eff}}$ measured by PFM on Au substrates

Materials	Thickness	$d_{33}^{\,\text{eff}}$ (pm/V)	Reference
BN	monolayer	0.04	15
MoS_2	monolayer	0.93	17
MoS_2	14.2−120 nm	0.35−0.44	15
MoS_2/BN	28.9−218 nm	1.03−4.44	15
WSe_2	monolayer	0.43	18
WSe_2	4−155 nm	0.29−0.57	this work
WSe_2/BN	21−40 nm	-0.12−-0.19	this work
Bi_2Se_3	37−110 nm	0.13−0.60	16
Bi_2Se_3/BN	160 nm	0.024	this work

Supporting Note 2 Phenomenological theory of $d_{33}^{\,\text{eff}}$

According to a phenomenological theory of the interface piezoelectricity arising from the built-in EF without external stresses, the longitudinal strain can be expressed as[13]

$$\varepsilon_{33} = M_{33}(E_{bi} + \Delta E)^2 = M_{33}E_{bi}{}^2 + 2M_{33}E_{bi}\Delta E + M_{33}\Delta E^2 \quad (3)$$

where M_{33} is the electrostriction coefficient, E_{bi} is the built-in EF, ΔE is an alternative EF induced by an applied AC voltage. The first term on the right side represents the static strain induced by the built-in EF; the second term describe the first-order harmonic strain induced by the AC voltage, which is the linear piezoelectric effect with the piezoelectric coefficient defined as $d_{33}^{\,\text{bi}} = 2M_{33}E_{bi}$. Due to the weak vdW forces, $d_{33}^{\,\text{bi}}$ is negative; the last term is the second-harmonic strain induced by the conventional electrostriction effect.

For vdW heterostructures, a previous work demonstrated a phenomenological piezoelectric model, where the piezoelectric coefficient is linearly related to the total polarization, P_3^{T}, which consists of three parts: the built-in EF induced polarization in vdW gaps, P_3^{bi}; the charge-transfer induced polarization in vdW material layers, P_3^{ml}; the intrinsic polarization in piezo/ferroelectric materials, P_3^{int}.[14] The strain induced by P_3^{bi} corresponds to the second term in Equation S3. For Bi_2Se_3/BN, P_3^{int} is zero. We modify P_3^{ml} by taking the polarization induced by the symmetry-breaking and the

lattice strain into consideration. In addition, we find from our simulation results that the flexoelectricity-induced polarization also plays a major role in the overall piezoelectricity. Then, we expand the piezoelectric model by taking flexoelectricity into account. Thus, the overall *d*33 eff coefficient for vdW heterostructures can be written as *d*33 eff = *d*33 bi + *d*33 fle + *d*33 ml, where *d*33 bi describes the interface piezoelectricity deriving from the built-in EF, *d*33 fle reflects the effective piezoelectricity stemming from flexoelectricity, and *d*33 ml represents the piezoelectricity due the polar structure in WSe_2 or Bi_2Se_3 layers caused by the symmetry breaking, the lattice strain, and the charge transfer.

References

(1) G. Kresse, J. Furthmü, Efficient iterative schemes for *ab initio* total-energy calculations using a plane-wave basis set. *Phys. Rev. B* **1996**, *54*, 11169.

(2) J. P. Perdew, K. Burke, M. Ernzerhof, Generalized gradient approximation made simple. *Phys. Rev. Lett.* **1996**, *77*, 3865.

(3) R. Gillen, J. Robertson, J. Maultzsch, Indirect doping effects from impurities in MoS_2/h-BN heterostructures. *Phys. Rev. B* **2014**, *90*, 075437.

(4) S. Xu, Y. Han, X. Chen, Z. Wu, L. Wang, T. Han, W. Ye, H. Lu, G. Long, Y. Wu, et al, van der Waals epitaxial growth of atomically thin Bi_2Se_3 and thickness-dependent topological phase transition. *Nano Lett.* **2015**, *15*, 2645-2651.

(5) I. S. Novikov, K. Gubaev, E. V. Podryabinkin, A. V Shapeev, The MLIP package: moment tensor potentials with MPI and active learning. *Mach. Learn.: Sci. Technol.* **2021**, *2*, 025002.

(6) B. Mortazavi, E. V. Podryabinkin, I. S. Novikov, T. Rabczuk, X. Zhuang, A. V Shapeev, Accelerating first-principles estimation of thermal conductivity by machine-learning interatomic potentials: A MTP/ShengBTE solution. *Comput. Phys. Commun.* **2021**, *258*, 107583.

(7) S. Plimpton, Fast parallel algorithms for short-range molecular dynamics. *J. Comput. Phys.* **1995**, *117*, 1-19.

(8) M. J. Frisch, G. W. Trucks, H. B. Schlegel, G. E. Scuseria, M. A. Robb, J. R.

Cheeseman, G. Scalmani, V. Barone, G. A. Petersson, H. Nakatsuji, et al, *GAUSSIAN 16 Revision B.01*, Gaussian Inc. Wallingford, CT, **2016**.

(9) S. Grimme, J. Antony, S. Ehrlich, H. Krieg, A consistent and accurate *ab initio* parametrization of density functional dispersion correction (DFT-D) for the 94 elements H-Pu. *J. Chem. Phys.* **2010**, *132*, 154104.

(10) B. Javvaji, B. Mortazavi, X. Zhuang, T. Rabczuk, Exploring tensile piezoelectricity and bending flexoelectricity of diamane monolayers by machine learning, *Carbon* **2021**, *185*, 558–567.

(11) B. Javvaji, X. Zhuang, T. Rabczuk, B. Mortazavi, Machine-learning-based exploration of bending flexoelectricity in novel 2D van der Waals bilayers. *Adv. Energy Mater.* **2022**, *12*, 2201370.

(12) C. J. Brennan, R. Ghosh, K. Koul, S. K. Banerjee, N. S. Lu, E. T. Yu, Out-of-plane electromechanical response of monolayer molybdenum disulfide measured by piezoresponse force microscopy. *Nano Lett.* **2017**, *17*, 5464–5471.

(13) M.-M. Yang, Z.-D. Luo, Z. Mi, J. Zhao, S. P. E, M. Alexe, Piezoelectric and pyroelectric effects induced by interface polar symmetry. *Nature* **2020**, *584* (7821), 377-381.

(14) Y. Chen, Z. Tang, H. Shan, B. Jiang, Y. Ding, X. Luo, Y. Zheng, Enhanced out-of-plane piezoelectric effect in In_2Se_3/transition metal dichalcogenide heterostructures. *Phys. Rev. B* **2021**, *104* (7), 075449.

(15) Q. Liu, V. K. Choyal, J. E. Morris, T. Rabczuk, X. Jiang, X. Zhuang, Largely enhanced out-of-plane electromechanical coupling effects in two-dimensional molybdenum-disulfide/boron-nitride heterostructures. *Nano Res.* **2025**, *18* (1), 94907050.

(16) Q. Liu, S. S. Nanthakumar, B. Li, T. Cheng, F. Bittner, C. Ma, F. Ding, L. Zheng, B. Roth, X. Zhuang, Converse flexoelectricity in van der Waals (vdW) three-dimensional topological insulator nanoflakes. *J. Phys. Chem. C* **2024**, *128* (38), 16265-16273.

(17) C. J. Brennan, R. Ghosh, K. Koul, S. K. Banerjee, N. Lu, E. T. Yu, Out-of-plane electromechanical response of monolayer molybdenum disulfide measured by

piezoresponse force microscopy. *Nano Lett.* **2017**, *17* (9), 5464-5471.

(18)C. J. Brennan, K. Koul, N. Lu, E. T. Yu, Out-of-plane electromechanical coupling in transition metal dichalcogenides. *Appl. Phys. Lett.* **2020**, *116* (5), 053101.
